# Rigiscan Evaluation of Men with Diabetes Mellitus and Erectile Dysfunction and Correlation with Diabetes Duration, Age, BMI, Lipids and HbA1c

**DOI:** 10.1371/journal.pone.0133121

**Published:** 2015-07-17

**Authors:** Daniel Peter Andersson, Urban Ekström, Mikael Lehtihet

**Affiliations:** 1 Department of Medicine, Karolinska University Hospital and Karolinska Institutet, Huddinge, Sweden; 2 Department of Medicine and Centre for Andrology and Sexual Medicine, Karolinska University Hospital and Karolinska Institutet, Huddinge, Sweden; Medical University Innsbruck, AUSTRIA

## Abstract

**Objective:**

This study aimed to investigate differences between patients with type 1 and type 2 diabetes mellitus with erectile dysfunction (ED) evaluated with Rigiscan and if there were a correlation to age, duration of diabetes, BMI, sex hormones, lipids and HbA1c.

Research design and methods: A retrospective study on patients with type 1 diabetes (n=15), type 2 diabetes (n=17) and a control group (n=31) that underwent Rigiscan examination for ED. Age, BMI, blood pressure, sex hormones, lipids and HbA1c were recorded and analyzed between groups.

**Results:**

Diabetes duration and HbA1C did not correlate with Rigiscan outcome. Rigiscan measures did not differ between patients with type 1 diabetes and control subjects besides from fewer erectile episodes (p<0.01) and lower tumescence activity units in base (p<0.05). By contrast, patients with type 2 diabetes differed significantly with respect to RigiScan parameters both in comparison with the type 1 diabetic patients and the control group. BMI had a strong correlation to number of erectile episodes, duration of erection, duration of erection > 60 % and rigidity activated unit (RAU) in tip and base. Age and HDL-cholesterol had a significant correlation with number of erectile episodes during night (p <0.05).

**Conclusion:**

Our results indicate that erectile dysfunction in men with diabetes differ between type 1 and type 2 diabetes patients. Neither diabetes duration nor HbA1C correlated to grade of erectile dysfunction among patients with diabetes. Increased BMI might be an explanation to the increased rate of erectile dysfunction seen in patients with type 2 diabetes.

## Introduction

Erectile dysfunction (ED), here defined as the inability to develop or maintain an erection of the penis during sexual activity, is a common finding among men with an age-standardized prevalence of around 40% [1]. Previous studies have shown that ED is a common finding in patients with diabetes regardless of insulin-dependence status and affect patients with diabetes 10–15 years earlier than the general population [2, 3]. However, some of these previous studies have several limitations. Type 1 and type 2 diabetes are two different diseases. A common finding among patients with type 2 diabetes is comorbidity with hypertension, hyperlipidemia and obesity; this is more uncommon among patients with type 1 diabetes. By introduction of more individual treatments for the diabetes disease and active treatment of hyperlipidemia and hypertension [4] complication rates in both groups have decreased [5, 6]. It is still a matter of controversy whether type 1 diabetes patients with ED have ED secondary to diabetes and decreased metabolic control [7] or if they, like other men, have ED secondary to cigarette smoking [8]or other multifactorial reasons [9]. One of the most reliable tools to diagnose ED and to differentiate psychogenic from organic cases is to monitor nocturnal penile tumescence and rigidity (NPTR) using the RigiScan device. The aim of this study was to retrospectively analyze ED evaluated with Rigiscan in men with type 1 and type 2 diabetes, and the impact of sex hormones, age, duration of diabetes, testosterone, BMI, HbA1c and lipids. We also wanted to investigate if there are special patterns of NPTR records in patients with diabetes vs. non diabetic.

## Research design and methods

### Subjects

This retrospective study was carried out from patients that underwent Rigiscan at department of Andrology and Sexual medicine at Karolinska University Hospital during the time period 2005 jan 1 to 2014 dec 31. A total of 394 patients were evaluated during this time period. During the same time period we investigated patients that also had the diagnose diabetes mellitus type 1 and 2 and underwent Rigiscan device. By using the International Classification of Diseases (ICD) diagnosis E10 and E11 we found 15 patients with type 1 diabetes, and 17 patients with type 2 diabetes that had been evaluated for erectile dysfunction with Rigiscan at our department and that fulfilled the inclusion and exclusion criteria.

Patients were included in the study if they in the medical history had ED of greater than 3-month duration and not could complete sexual intercourse due to poor erection and excluded if they have one of the following: neurologic disease, genital or spinal cord injuries, morbid obesity (body mass index> 35 kg/m^2^), severe heart disease, penile fibrosis, uncontrolled hypertension (Uncontrolled hypertension was defined as an average systolic blood pressure ≥140 mmHg or an average diastolic blood pressure ≥90 mmHg, among those with hypertension), treatment with testosterone or derivate or hypogonadism. All men included in the study underwent a thorough ED history taking by experienced physicians. The physical examination consisted of general, genital, neurologic, and urologic examinations. Through a self-made computer program non-diabetes controls were selected at random and included if the fulfilled inclusion and exclusion criteria. All patients underwent blood chemistry testing including serum testosterone, prolactin, lipids and HbA1c levels.

In table 1 study characteristics of the participants are included. The time between blood test and Rigiscan analyses did not exceed four weeks for any of the participants. The study was approved by the local committee on ethics at Karolinska Institutet. All records were anonymized and de-identified prior to analysis.

**Table 1:** Baseline clinical characteristics of participants at group level. Values reported are mean ± standard deviation (SD). Mann-Whitney U-test) was used to test the differences between the groups. Data was considered statistically significant at *P* < 0.05.

	Control (A)	Type 1 diabetes (B)	Type 2 diabetes (C)	A vs B	A vs C	B vs C
n	31	15	17			
Age (years)	49±5	45±8	51±7	0.10	0.16	<0.05
Duration of diabetes (years)		28±7	9±5			<0.001
BMI (kg/m^2^)	28.2±3.4	25.6±3.6	30.5±4.4	0.10	0.11	<0.01
Systolic blood pressure (mmHg)	130.8±14.6	128.6±10.3	131±10.7	0.79	0.72	0.64
Diastolic blood pressure (mmHg)	80.9±7.9	77.4±9.6	78.1±8.0	0.30	0.50	0.76
HbA1c(mmol/mol)	35.4±4.4	69.00±11.2	65.3±8.8	<0.001	<0.001	0.28
S-LH (U/L)	3.35±1.5	3.8±1.7	3.7±2.3	0.20	0.87	0.31
S-Testosterone (nmol/L)	14.2±4.1	16.5±3.9	14.1±2.6	<0.05	0.47	0.088
S-SHBG (nmol/L)	37.2±14.6	50.3±14.8	39.4±16.6	<0.01	0.49	0.11
S-Prolactin (ug/L)	7.8±1.7	9.0±2.7	9.3±2.5	0.12	<0.05	0.74
S-Cholesterol (mmol/L)	5.3±1.1	4.8±1.0	5,4±1.6	0.16	0.92	0.37
S-HDL (mmol/L)	1.2±0.3	1.6±0.6	1.1±0.2	0.067	0.053	<0.001
S-LDL (mmol/L)	3.4±0.9	2.9±1.0	3.2±1.1	0.14	0.76	0.49
S-Triglycerides (mmol/L)	1.3±0.5	1.0±0.6	2.5±2.4	<0.05	<0.05	<0.001

### Rigiscan analysis

The RigiScan Plus software 4.0 was used in this study. Summary statistics provided by the software, is able to recognize erectile activity as an event following a 20% increase in the base loop circumference persisting for at least 3 min and also include the number of events detected, and integrated time intensity area measures of tumescence (tumescence activity units [TAU]) and rigidity (rigidity activity units [RAU]). These two units of measurement, RAU and TAU, were developed to facilitate the interpretation of the time-dependent nature of rigidity and tumescence. RAU represents the product of the minutes spent at a given rigidity level. The rigidity level is expressed in decimal form. This value is calculated on a point-by-point basis and summarized for the entire erectile event. Similarly, TAU represents the time of duration of an erectile event multiplied by the percentage increase of circumference (expressed as a decimal) over the estimated baseline tumescence.

Rigiscan analysis was performed during minimum two nights sleep with minimum 5 hours sleep per night and with general recommendations to avoid alcohol, chemical sleep aids or phosphodiesterase type 5 (PDE-5) inhibitors for two nights before the test. After each monitoring period all data were transferred to a personal computer and were analyzed with RigiScan Plus software version 4.0. Sessions less than five hour’s duration were excluded from further analyses. Erectile activity during sleep was measured by determining the following parameters: number of erectile episodes, duration of erectile episodes (min), duration of rigidity > 60%, increase of circumference in stimulated vs. unstimulated state (%) and TAU–RAU values. The best results from the two or three-night registrations were used for analyses.

### Assays

Testosterone was measured with a chemiluminescent immunoassay for quantitative determination of total testosterone level in human serum and plasma using the Access Immunoassay System (Beckman Coulter). The detection rate range was between 0.35–55.5 nmol/L). The intra- and inter-assay CV for testosterone were less than 5 percent in both cases. Sexual hormone binding globulin (SHBG) and prolactin were measured with a paramagnetic particle chemiluminescent immunoassay (Beckman Coulter, Inc). The intra-and inter-assay variation for SHBG was 4.0 and 5.5 percent respectively, and for prolactin 3.5 and 5.0 percent. Total cholesterol, High-density lipoprotein (HDL)-cholesterol and HbA1c were measured by the routine chemistry accredited laboratory at the Karolinska University Hospital. LDL-cholesterol was calculated according to the Friedewald formula [10]. All samples were analyzed according to the manufacturer’s advice at the Department of clinical chemistry, Karolinska University Hospital.

### Statistical analyses

Mann-Whitney U-test) was used to test the differences between the groups. Correlation was tested with a Spearman rank correlation coefficient model. Data was considered statistically significant at *P* < 0.05. Values reported are mean ± standard deviation (SD). Statistical analyses were performed using Statistica, Statsoft version 10.0 (Tulsa, OK, USA).

## Results

### Clinical characteristics

The clinical characteristics of the 62 men who were included in the study are shown in Table 1. The group of men enrolled in this study did not differ significantly in systolic or diastolic blood pressure. However, there was a significant difference between age and BMI in patients with type 1- and type 2 diabetes. Patients with type 2 diabetes were older and had higher BMI (Table 1). Patients with type 1 diabetes had longer known diabetes duration, p< 0.001.

### Metabolic control as reflected by HbA1c and lipids

HbA1c levels were similar in the patients with type 1 and type 2 diabetes (69±11 mmol/mol and 65±8.8 mmol/mol, respectively). Total cholesterol and LDL did not differ between diabetes patients and control group, Patients with type 1 diabetes had the lowest triglyceride levels even compared with the control group. Patients with type 2 diabetes had the lowest HDL level, p<0.01 vs. type 1 diabetes, Table 1.

### Sex hormones

Patients with type 1 diabetes had higher level of total testosterone vs. the control group. However, the levels of total testosterone in both groups were in the normal range. Patients with type 1 diabetes also had higher level of SHBG which could be secondary to poorer metabolic control with hypo-insulinemic condition vs. healthy controls and patients with type 2 diabetes. This is supported by LH value among the different groups, Table 1. The bioavailability testosterone level did not differ between the groups, data not shown. Prolactin level between groups did not differ significantly, Table 1.

### Rigiscan parameters

The Rigiscan characteristics of the patients are described in Table 2. As group there was a difference in number of nightly erectile episodes between control group and both type 1 and 2 diabetes patients and also a significant difference between type 1 and type 2 diabetes patients. Duration of erectile activity and duration of rigidity over 60% did however not differ between control groups and type 1 diabetes patients. A significant difference was seen between patients with type 1 and type 2 diabetes. Rigidity activated unit (RAU) and tumescence activated unit (TAU) both in tip and base were significant different between control group and patients with type 2 diabetes, table 2. A small but significant difference in TAU base was seen in type 1 diabetes group vs. control group, table 2. Also the circumference stimulated vs unstimulated between the three different groups of patients did significantly difference between control group and patients with type 1 diabetes and between control group and patients with type 2 diabetic. No significant difference was seen between type 1 diabetes patients and control group, Table 2.

**Table 2:** Comparison of Rigiscan parameters. Values reported are mean ± SD. Mann-Whitney U-test) was used to test the differences between the groups. Data was considered statistically significant at *P* < 0.05.

	Control (A)	Type 1 diabetes (B)	Type 2 diabetes (C)	A vs B	A vs C	B vs C
N	31	15	17			
Number of erectile episodes	4.8±1.4	3.4±1.2	2.9±1.5	<0.01	<0.001	0.31
Duration of erectile episodes (min)	132.7±53	104.4±45	73±44.9	0.078	<0.001	<0.05
Duration of rigidity > 60%	61.1±51.9	53.6±35.9	26.7±23.0	0.91	<0.05	<0.05
RAU tip	46.2±23.6	39.3±17.5	25.6±21.9	0.27	<0.01	<0.05
RAU base	63.8±26.9	58.7±27.5	36.1±25.5	0.65	<0.01	<0.05
TAU tip	31.3±16.7	21.3±9.6	20.1±15.0	0.067	<0.05	0.75
TAU base	51.4±23.7	34.3±20.0	31.9±23.2	<0.05	<0.01	0.43
Circumference tip (%)	32.4±8.0	29.4±7.1	22.5±9.3	0.28	<0.01	<0.05
Circumference base (%)	37.0±10.6	34,2±4.3	18.0±8.4	0.28	<0.01	<0.05

### Effect of age, diabetes duration, total testosterone, BMI and cholesterol on erectile dysfunction evaluated by Rigiscan

In this study age was significantly correlated to number of erectile episodes and time duration of erectile episodes during night, but rigidity over 60% did not decrease with age. We could not find any correlation with duration of diabetes. Subgroup analyses of type 1 and type 2 diabetes patients *per se* did not give other results compared to overall analysis (data not shown). We could not see any significant correlation between total testosterone levels and Rigiscan parameters. Although not significant, a low grade of correlation was seen between number of erections and total testosterone level, however none of the patients were having clinically low testosterone level or sign of hypogonadism at medical examination. BMI had significant correlation with most of the Rigiscan parameters (Table 2), except TAU in tip and base. There was a positive correlation with overall HDL level and number and duration of erectile episodes and RAU and TAU in both base and tip but no significant correlation was seen between HDL and duration of erectile rigidity over 60% (Table 3). Neither total cholesterol levels nor LDL-cholesterol had a significant impact on ED. We could not find any correlation among the diabetes patients HBA1c level and Rigiscan parameters, data not shown.

**Table 3:** Correlation between Rigiscan parameters was tested with a Spearman rank correlation coefficient model. Values reported are mean ± SD. Data was considered statistically significant at *P* < 0.05.

	Age (year)		Duration of diabetes (year)		Total Testosterone (nmol/L)		BMI overall (kg/m^2^)		S-Cholesterol (mmol/L)		S-HDL (mmol/L)		S-LDL (mmol/L)	
	p	r	P	r	P	r	p	r	p	r	p	r	p	R
Number of erectile episodes	<0.05	-0.31	0.73	-0.06	0.06	0.24	<0.05	-0.31	0.20	0.16	<0.05	0.26	0.13	0.19
Duration of erectile episodes (min)	<0.05	-0.32	0.61	0.092	0.63	0.060	<0.01	-0.33	0.645	0.05	<0.01	0.39	0.78	0.035
Duration of rigidity > 60%	0.28	-0.13	0.22	0.21	0.31	-0.13	<0.01	-0.37	0.26	0.14	0.22	0.15	0.27	0.14
RAU tip	0.27	-0.14	0.23	0.22	0.67	-0.05	<0.01	-0.36	0.13	0.18	<0.05	0.29	0.36	0.12
RAU base	0.20	-0.16	0.14	0.26	0.79	0.034	<0.05	-0.30	0.14	0.18	<0.01	0.34	0.23	0.15
TAU tip	0.99	0.0010	0.93	-0.016	0.61	-0-064	0.17	-0.24	0.15	0.18	<0.05	0.28	0.17	0.18
TAU base	0.99	-00014	0.79	0.047	0.34	-0.12	0.60	-0.09	0.12	0.19	<0.01	0.35	0.52	0.083

## Discussion

Erectile dysfunction is a symptom causing a substantial decrease in quality of life [11]. To our knowledge, this is the first study to investigate to what extent type of diabetes, diabetes duration and HbA1C have an impact on non-psychogenic erectile dysfunction evaluated with Rigiscan. In contrast to our original hypothesis and what has been shown in several studies evaluating erectile dysfunction by questionnaires [12], type 1 diabetes does not seem to have negative effects on erectile function when comparing subjects with or without type 1 diabetes. However, compared to control subjects, type 2 diabetes correlates with erectile dysfunction, which could probably be explained by an increased BMI seen in this group of patients. Interestingly, neither duration of diabetes nor HbA1C levels effected erectile function in patients with type 1 or type 2 diabetes. In both control subjects and patients with type 1 or type 2 diabetes we saw a positive correlation between HDL levels and erectile function, but not total cholesterol or LDL cholesterol. In a placebo-controlled study evaluating the effect of atorvastatin on response to sildenafil in subjects with ED, a treatment and lowering of LDL cholesterol and increase of HDL cholesterol were accompanied with decrease in ED [13]. Whether LDL cholesterol and/or HDL cholesterol levels have an impact on ED cannot be determined by our study.

Several studies have shown that an expectation of erectile dysfunction often results in ED and difficulties in treatment of this symptom [14]. A common belief among both physicians and the public is that diabetes is a common cause of erectile dysfunction. The results in our study challenge this belief in patients with type 1 diabetes. This new knowledge can potentially have a large clinical impact on subjects with type 1 diabetes and their quality of life. If a physician can tell a patient that he does not *per se* have an increased risk of erectile dysfunction because of his type 1 diabetes, the risk of psychogenic erectile dysfunction in this group should not be higher than in the general population. This knowledge could lead to a both a decreased risk of developing and an enhanced chance of treating erectile dysfunction.

### Strengths and limitations

The major strength with our study is that it, in contrast to previous studies on subjects with diabetes, compares erectile function evaluated with the unbiased Rigiscan measurement and also compares patients with type 1 diabetes and type 2 diabetes with control subjects.

Admittedly, our study has several limitations. Although Rigiscan has been widely used in several studies to evaluate erectile dysfunction by measuring number and quality of erections during sleep, it has been questioned by some due to its role as a golden standard for ED diagnosis [15]. In the present study we examined the patients as groups and not at individual level. Therefore we didn’t use standard criteria for erectile dysfunction, as previously described [9, 16]. Our main purpose was therefore not to differentiate psychogenic from organic ED. For an accurate diagnostic approach, NPTR should be combined with other conventional diagnostic methods [17]. This study should be considered to be a pilot study due to the limited number of subjects. Furthermore, the cross-sectional design of our study makes it impossible to draw any conclusions regarding causality. Larger prospective studies with repeated measurements of HbA1C, blood lipids and including the use of the same or other objective assessments of erectile dysfunction would be of great value.

## Conclusion

This study shows that subjects with type 1 diabetes, in contrast to subjects with type 2 diabetes, does not have an increased risk of developing erectile dysfunction compared to the general population. Although this needs to be confirmed in larger longitudinal studies, the results in this study can have a great clinical impact. With the results from this pilot study, physicians may say to their patients with type 1 diabetes that the association between type 1 diabetes and erectile dysfunction is under investigation. This may lead to a decreased risk of developing psychogenic erectile dysfunction and an enhanced chance of successful treatment of erectile dysfunction.

## Supporting Information

S1 Dataset Original dataset used for all calculations. (XLS)
